# Supplementary material for: Embryonic Thermal Manipulation Affects Body Performance Parameters and Cecum Microbiome in Broiler Chickens in Response to Post-Hatch Chronic Heat Stress Challenge
Source: Animals (Basel). 2025 Jun 6;15(12):1677. doi: 10.3390/ani15121677 (PMC12189558; doi:10.3390/ani15121677)
Supplement: Supplementary file 1 [file animals-15-01677-s001.zip › animals-3599125-supplementary.pdf]

animals

MDPI

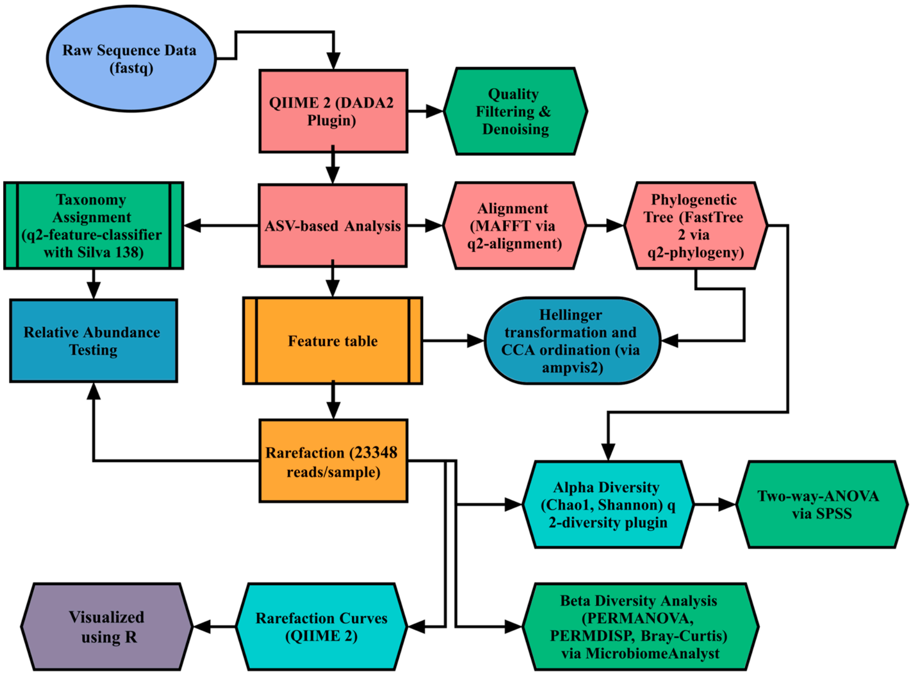

Figure S1. Summary of the bioinformatic analysis carried out in this study. ASV: amplicon sequencing variant, CCA: Canonical Correspondence Analysis, PERMANOVA: Permutational Multivariate Analysis of Variance, PERMDISP: permutational analysis of multivariate dispersions.

Rarefaction Curves Analysis

Figure S2 presents the rarefaction curves, which plot the Shannon index as a function of sequencing depth. Notably, all groups' curves plateaued, suggesting that enough sequencing depth was attained to capture the diversity within each group.

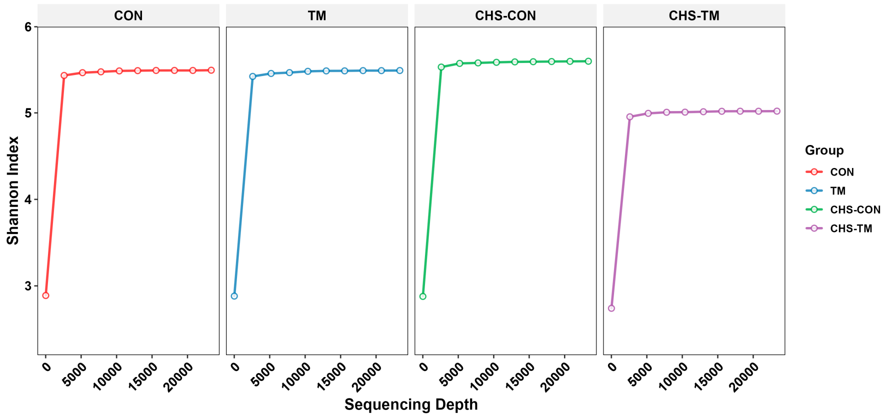

Figure S2. The curves approach a plateau, indicating that the sequencing depth was sufficient to capture the most microbial diversity in all groups.

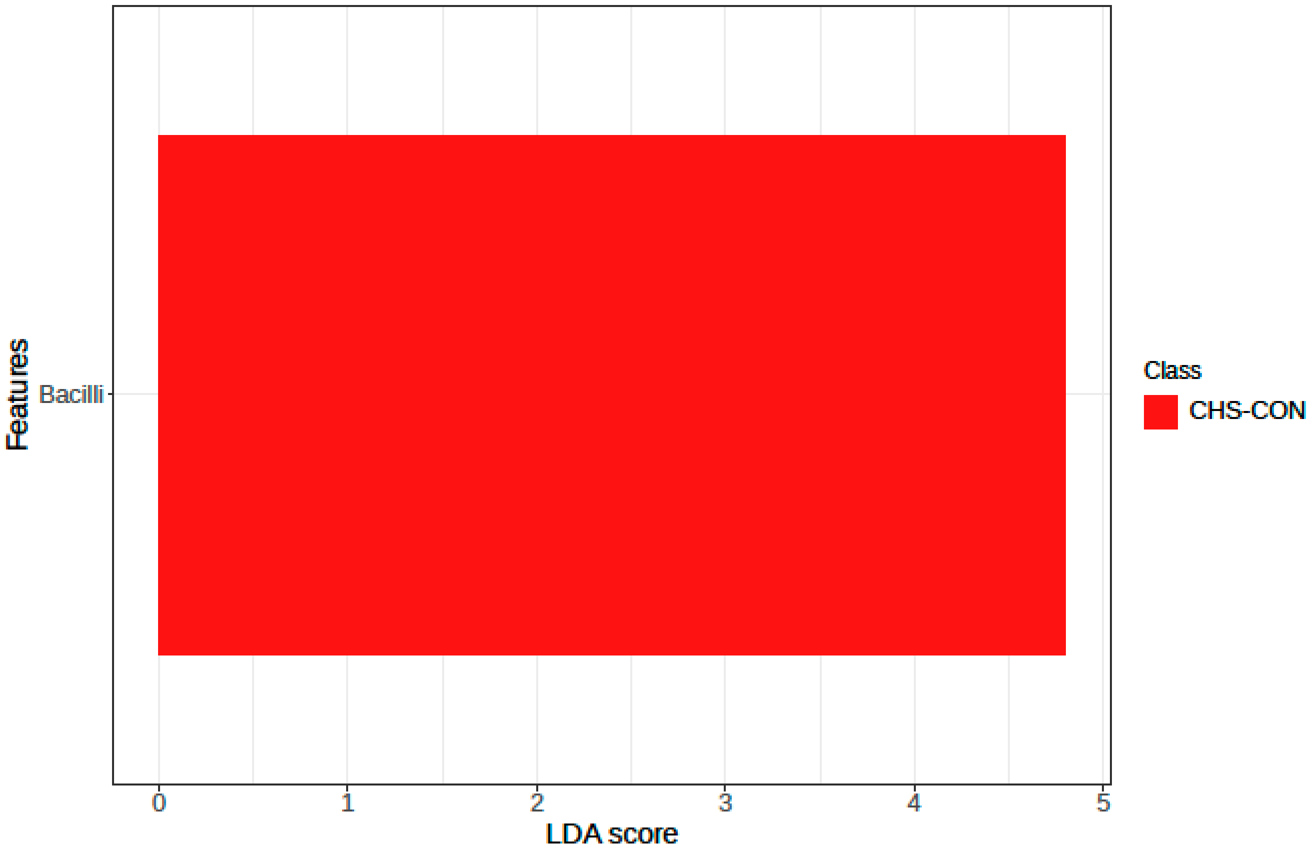

Figure S3. Represent Linear Discriminant Analysis (LDA) score plot at class level, highlighting the most discriminative features (bacterial class) between treatment groups. The LDA scores were calculated using the LEfSe (Linear discriminant analysis Effect Size) algorithm to identify taxa with significant differences in relative abundance across groups. Taxa with an LDA score above threshold 3.0 were considered significantly enriched. The horizontal bars represent the effect size of each taxon, with longer bars indicating greater discriminatory power. P value < 0.05.

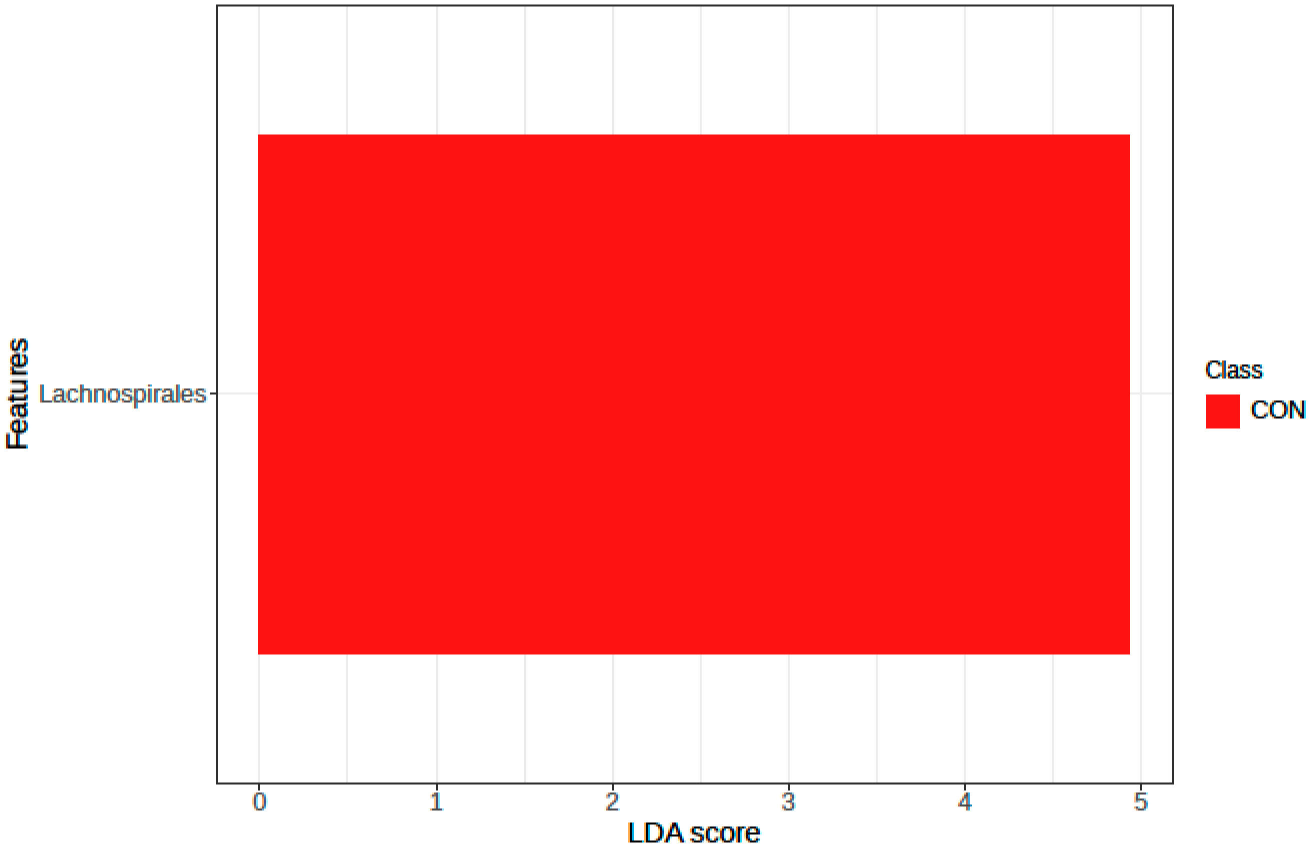

Figure S4. Represent Linear Discriminant Analysis (LDA) score plot at order level.

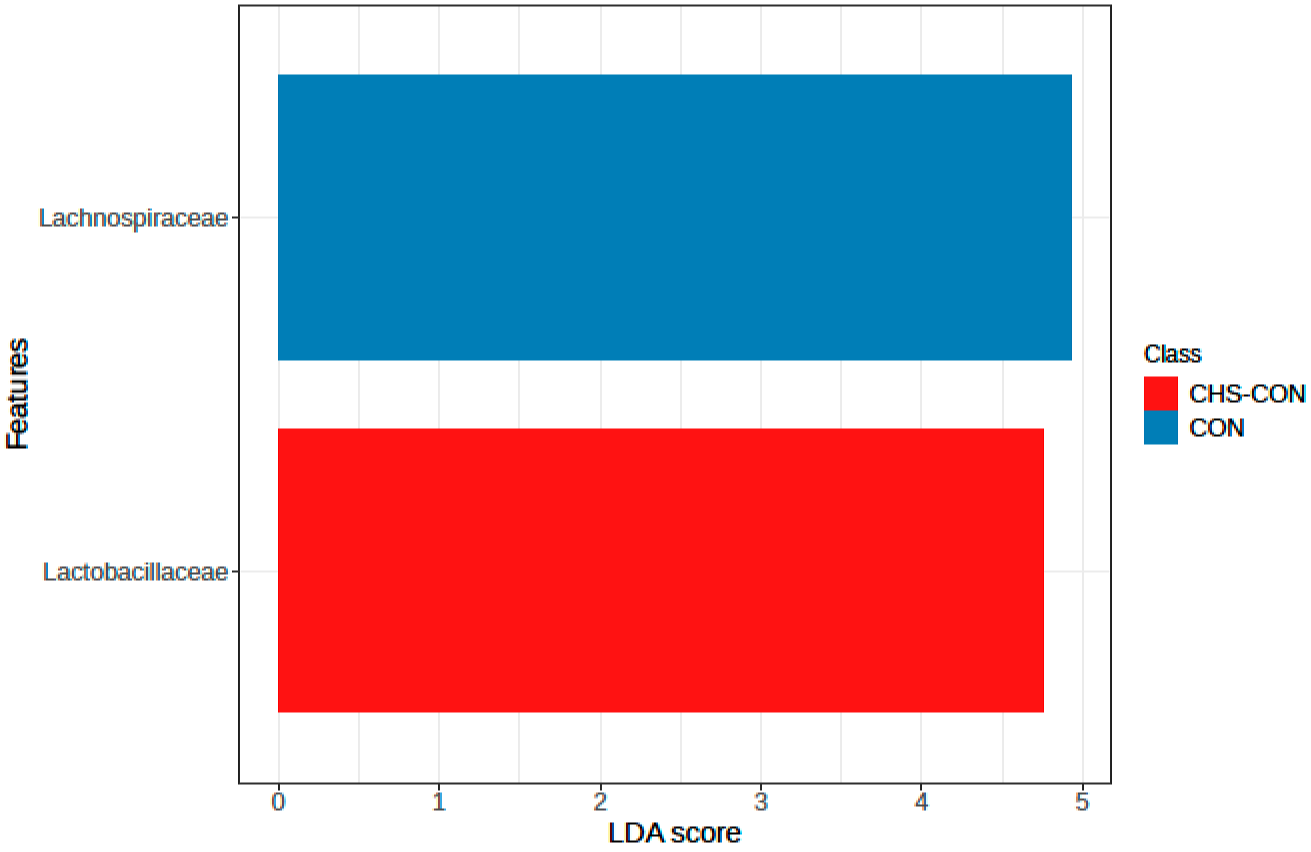

Figure S5. Represent Linear Discriminant Analysis (LDA) score plot at family level.

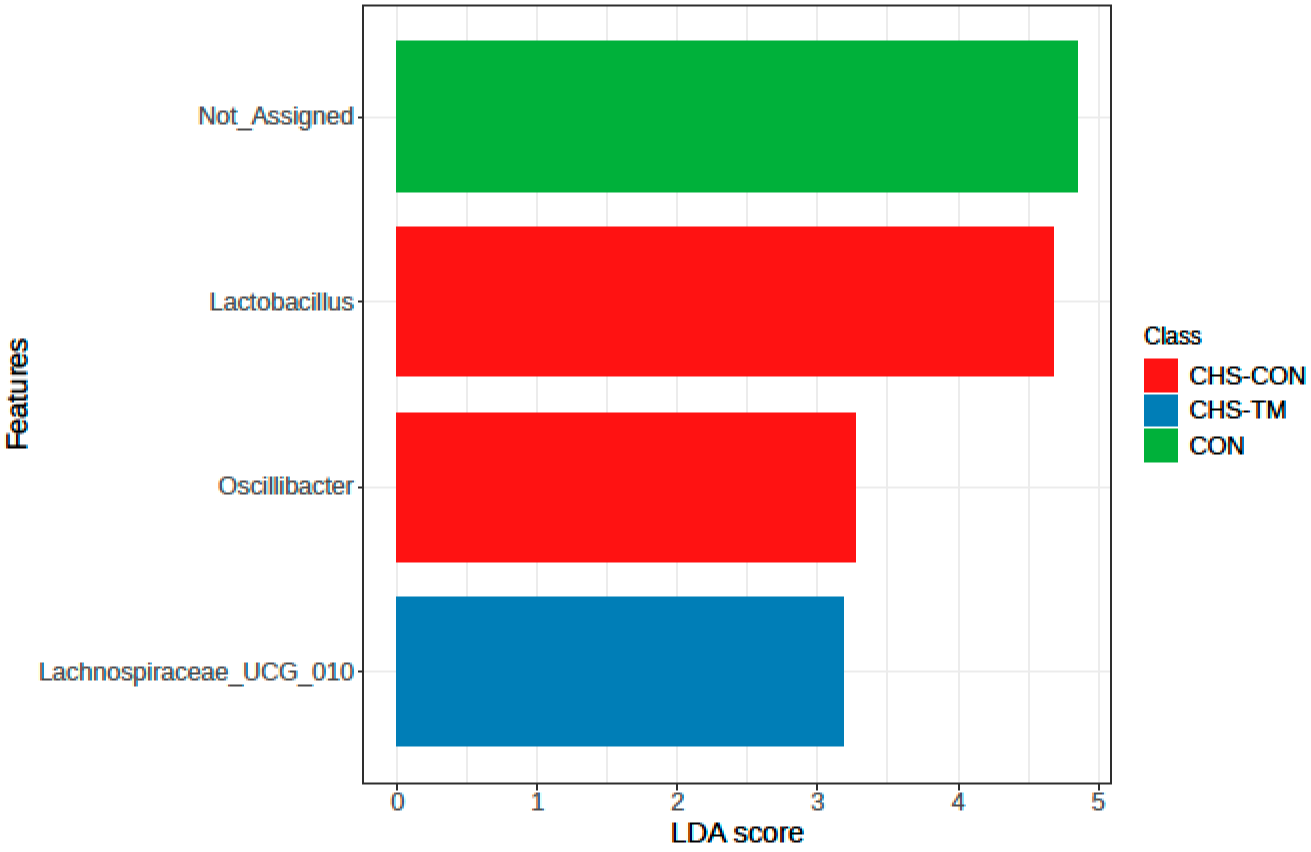

Figure S6. Represent Linear Discriminant Analysis (LDA) score plot at genus level.

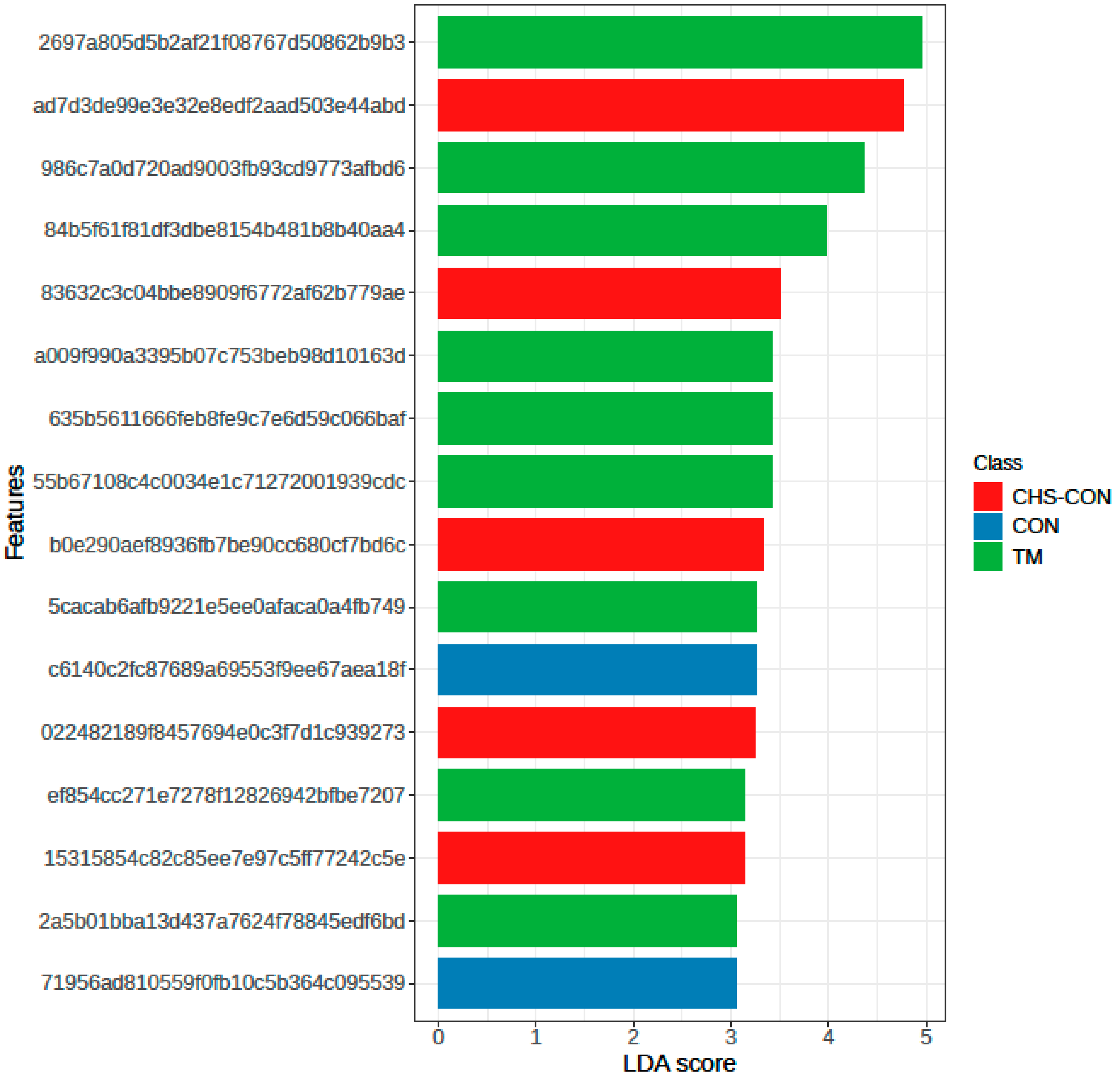

Figure S7. Represent Linear Discriminant Analysis (LDA) score plot at feature level. Highlighting the distinctive bacterial taxa associated with each group.

Population diversity is a crucial feature of a microbiome community closely linked to its environment. The shift in diversity could be a sign that the animal body's environment has changed (from a healthy to a diseased state, for example) or that certain events have disturbed the environment (such as using antibiotics or a shift in the immune system). Therefore, examining the diversity of the gut microbiota and identifying host and/or environmental factors that can alter the taxonomic composition is one of the primary objectives of microbiome community study (56, 57). The microbiome community typically has three types of diversity: alpha, beta, and gamma. The two most widely used diversity metrics in microbiome research are alpha and beta diversity (58). While beta-diversity measures compare the similarity of two or more communities, alpha-diversity is a broad word for metrics that characterize the species richness, evenness, or diversity within a sample (59, 60). In this study, the Chao1 index was used to assess community richness, while the Shannon index was employed to estimate community diversity. The Chao1 index provides a nonparametric estimate of taxonomic richness based on abundance (61), whereas Shannon's index H combines evenness and richness to evaluate taxonomic diversity (62).

Sequencing depth significantly impacts how accurately 16S rRNA sequencing measures microbial diversity. To address this, rarefaction is generally considered a valuable method to regulate unequal sequencing depth when analyzing alpha- and beta-diversity (63). Specifically, this technique ensures comparability by subsampling reads to a predetermined depth without replacement, standardizing library size across samples. However, excluded samples are those whose total number of reads falls below the required depth. In this study, rarefaction was carried out at a depth of 23348 reads per sample to ensure consistent sequencing coverage across all examined groups.
